# Enhancing Dermatological Diagnostics with EfficientNet: A Deep Learning Approach

**DOI:** 10.3390/bioengineering11080810

**Published:** 2024-08-09

**Authors:** Ionela Manole, Alexandra-Irina Butacu, Raluca Nicoleta Bejan, George-Sorin Tiplica

**Affiliations:** 1 2nd Department of Dermatology, Colentina Clinical Hospital, 020125 Bucharest, Romaniageorge.tiplica@umfcd.ro (G.-S.T.); 2 2nd Department of Dermatology, Carol Davila University of Medicine and Pharmacy, 050474 Bucharest, Romania; 3 Adobe Romania, 061331 Bucharest, Romania; raluca.ioan@gmail.com

**Keywords:** artificial intelligence, benign lesions, classification, malignant lesions, neural networks, transfer learning

## Abstract

**Background**: Despite recent advancements, medical technology has not yet reached its peak. Precision medicine is growing rapidly, thanks to machine learning breakthroughs powered by increased computational capabilities. This article explores a deep learning application for computer-aided diagnosis in dermatology. **Methods**: Using a custom model based on EfficientNetB3 and deep learning, we propose an approach for skin lesion classification that offers superior results with smaller, cheaper, and faster inference times compared to other models. The skin images dataset used for this research includes 8222 files selected from the authors’ collection and the ISIC 2019 archive, covering six dermatological conditions. **Results**: The model achieved 95.4% validation accuracy in four categories—melanoma, basal cell carcinoma, benign keratosis-like lesions, and melanocytic nevi—using an average of 1600 images per category. Adding two categories with fewer images (about 700 each)—squamous cell carcinoma and actinic keratoses—reduced the validation accuracy to 88.8%. The model maintained accuracy on new clinical test images taken under the same conditions as the training dataset. **Conclusions**: The custom model demonstrated excellent performance on the diverse skin lesions dataset, with significant potential for further enhancements.

## 1. Introduction

As the most common cancer in the world, skin cancer has increased dramatically over the last few decades. Skin cancer is divided by dermatologists into two major types: non-melanoma skin cancers (NMSCs) and melanoma, recognized for its aggressive behavior and risk of metastasizing. While occurring less frequently than NMSCs, melanoma is responsible for the majority of skin cancer mortality, pointing out the urgent need for accurate diagnosis [1].

The most common type of skin cancer is NMSC, which includes mainly basal cell carcinoma (BCC) and squamous cell carcinoma (SCC). BCC rarely metastasizes but is associated with significant morbidity in those cases that are left untreated or treated with delay. SCC, although more aggressive than BCC in terms of metastasis, usually responds well to treatment if detected and treated on time. Increased incidence of NMSCs has been associated with higher ambient UV, related to ozone depletion, and increased public awareness and screening activities [2].

Melanoma, although a relatively rare form of skin cancer, is associated with a highly metastatic behavior. Early detection is of extreme importance to achieving a high survival rate in effective melanoma management. The five-year survival rate for patients with early-stage melanoma is over 90%, but this figure decreases significantly with the higher stage at diagnosis [3].

Benign skin lesions, including actinic keratosis (AK), benign keratosis-like lesions, and melanocytic nevi, are widespread and typically non-malignant. Despite their benign behavior, clinical differentiation from their malignant counterpart is sometimes challenging, even for an experienced dermatologist. When distinguishing between malignant and benign diseases, this diagnostic challenge may result in either unnecessary invasive procedures or delayed interventions.

The differential diagnosis of skin lesions is a complex task that reflects a cumulative knowledge of their morphology, distribution, and evolution over time. For malignant and benign skin lesions, dermoscopy, a technique that permits improved visualization, has provided a great deal of diagnostic accuracy. However, dermoscopic analysis can be subjective and experience-dependent, which can result in variations in diagnosis and outcomes [4].

Early and accurate diagnosis of skin lesions is of utmost importance for treatment outcomes, especially when considering skin cancer. According to the World Health Organization, 1.5 million cases of skin cancers were reported in 2022, of which 330,000 were melanoma cases, leading to almost 60,000 deaths [5]. Therefore, the precise and timely diagnosis of skin lesions is essential, as it directly impacts patient management and prognosis. Although established diagnostic methods are effective, they can be subjective when using clinical evaluation as the main diagnostic tool. Such heterogeneity highlights the importance of moving toward diagnostic tools that are less subjective and more repeatable and scalable, which can also enhance early detection and improve clinical outcomes.

Today, the diagnostic landscape in dermatology is changing through the great impact of emerging technologies with depth in science, such as deep learning (DL) and artificial intelligence (AI). DL, a subset of machine learning (ML), has emerged as a powerful tool that is predicted to be a game changer in multiple fields of medicine, including dermatology. Computers and improved ML models can now solve hard, complicated diagnostic tasks with high accuracy. AI models, particularly when trained on large-scale databases, have the potential ability to be similar to or superior to dermatologists in skin lesion classification. Moreover, these AI systems have a major impact in providing standardization in diagnostic interpretations and in the reduction in the inter-observer variability and frequency of diagnostic errors [6].

This consistent, repeatable interpretation may revolutionize skin lesion diagnostics as AI becomes integrated into clinical practice. Further, such integration allows for enhanced distribution of dermatologic expertise to underserved regions, democratizing access to expert-level diagnosis and care. To accommodate the vast diversity of skin types and conditions we can expect to handle in clinical practice, a durable deployment of AI tools in dermatology may require extensive validation and continuous training on multiple datasets [7].

Up until recently, in dermatology, DL models have outperformed standard diagnostic approaches in several studies. For instance, research performed by Esteva et al. (2017) has demonstrated that convolutional neural networks (CNNs) can classify skin cancer with a level of competence comparable to dermatologists, using vast datasets of dermatoscopic images [8]. Similarly, Codella et al. (2017) reported that ensemble DL could remarkably improve melanoma detection in dermoscopy images [9]. In a more recent paper from Brinker et al. (2019), deep neural networks were found to outperform dermatologists after being trained on images of melanomas, indicating the potential of these models for clinical decision-making applications [10]. Moving forward, Liu et al. (2020) also reported an AI attempt to diagnose skin diseases with a differential diagnostic accuracy comparable to dermatologists [11]. These advances in AI are not only being transformed into plain research findings but they are also being manifested into practical application results that could make use of saving lives through real clinical operations. These echo the belief within the medical field that AI, specifically DL algorithms, can vastly improve both the accuracy and the consistency of diagnostic tests.

Nonetheless, as these advanced AI tools are being considered to be included in a clinic setting, significant challenges with deployment remain, driven by requirements in extensive training data and generalization across diverse patient populations. This also includes the validation process intended to demonstrate accuracy and reliability.

In this study, EfficientNetB3, an architecture that achieves state-of-the-art accuracy in image classification, was used. Developed by Tan and Le (2019), it is the ideal type of architecture for medical image analysis as it performs the best in benchmarks considering both size and computational efficiency [12]. The work presented in this article focuses on the usage of a custom model based on EfficientNetB3 that uses transfer learning to analyze skin lesions on an extended dataset. This study aims to bridge the gap between the rapid advancements in technology and clinical utility in a scalable and efficient fashion to improve diagnostic accuracy and speed in dermatology by applying advanced AI methods. The model was trained and validated across six classes of skin diseases, encompassing benign and malignant conditions such as melanoma, basal cell carcinoma, squamous cell carcinoma, actinic keratoses, benign keratosis-like lesions, and melanocytic nevi.

By integrating the latest AI methods, progress can be achieved, such as improving diagnosis or reducing clinicians’ workload, allowing more time for patient care and less for routine diagnosis. Combining AI strengths on the technical side with the nuanced understanding of skin pathology from expert dermatologists, we have created an interdependent mix that betters the accuracy and applicability of the diagnostic process.

There are several novel contributions that have been introduced to dermatological research and clinical practice; however, our custom model:

- Ranks among the top-performing models in the European region, indicating its potential to address regional medical challenges effectively.
- Achieves competitive results based on EfficientNetB3, demonstrating efficient utilization of limited training data.
- Enhances practical feasibility and cost-effectiveness of deployment due to its modest computational requirements.
- Shows robust performance with fewer images compared to models that achieve similar or better results with larger datasets.

The following part of this study is organized as follows: Section 2 details the training dataset, data preprocessing steps, the model architecture and hyperparameters, and techniques to combat overfitting, along with the training and validation processes. Section 3 presents the model’s performance. Section 4 interprets the results, highlighting their implications and limitations. Finally, the conclusion summarizes the findings and suggests directions for future research.

## 2. Methodology of Research

### 2.1. Description of the Training Dataset

**Figure 1:**
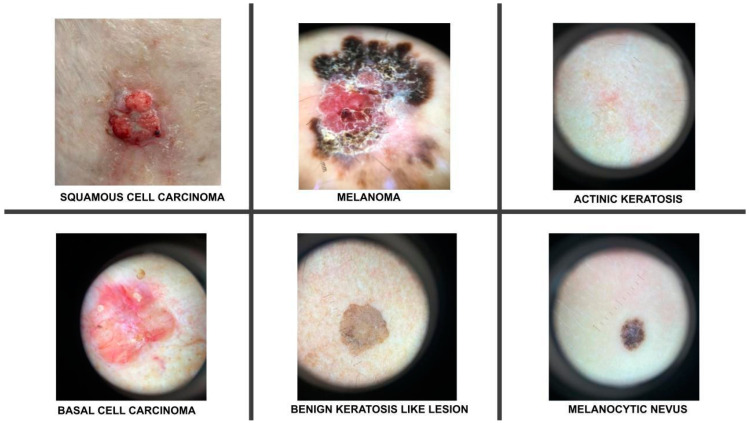
Examples of clinical and dermoscopic images used for training.

The primary dataset used for training was a proprietary combination of files collected from the authors’ collection of images and the International Skin Imaging Collaboration 2019 (ISIC 2019) archive [13]. The dataset used in this study comprised 8222 images, covering six categories of dermatological lesions. These categories include three malignant skin conditions—melanoma, BCC, and SCC—and three non-malignant conditions—AK, benign keratosis-like lesions, and melanocytic nevi (Figure 1).

The data were divided into training, validation, and testing sets, with the training set consisting of 80% of the data (6578 images) and the validation and test sets containing 20% (1644 images). Table 1 includes the distribution of all images across the disease categories.

Dermoscopic and close-up images were included in the dataset, ensuring that each image consistently belonged to one of the specified categories. The EfficientNetB3 model was initially trained on the ImageNet image database [14] using a resolution of 300 × 300 pixels, keeping the RGB color model. Consequently, all images in this project were resized to 300 × 300 pixels. This resizing process maintained the defining properties and shapes of the lesions, ensuring no distortion occurred. Additionally, this standardization reduced the dataset’s size, enabling faster model training.

### 2.2. Data Preprocessing

Before feeding images into the neural network model, the following preprocessing steps were applied to optimize model performance. This preparation ensured efficient and effective learning:

- Resizing: Images were resized to 300 × 300, ensuring uniformity in input sizes.
- Normalization: Pixel values were scaled to the [0, 1] interval to reduce data discrepancies and aid training convergence.
- Mean Subtraction and Standardization: Each pixel’s value had the dataset’s mean subtracted and was then divided by the standard deviation to normalize the data further, enhancing model convergence.
- Data Augmentation: This technique creates new images by modifying existing ones. We employed mirroring, translation, rotation, scaling, brightness adjustment, and noise addition to augment the existing pictures [15]. These augmented images were then added to the categories with less data, thereby balancing the training dataset.

### 2.3. The Usage and the Architecture of the Model

The EfficientNet neural network class of models, first proposed by Tan and Le [12], is a family of image classification models known for its feature extraction capabilities that achieved very good accuracy while being 8.4× smaller and 6.1× faster on inference than the best existing ConvNet neural networks [16]. The decision to use a model based on EfficientNetB3 was driven by its effective balance between classification performance and resource efficiency. Compared to larger models like EfficientNetB4, B5, and B6, EfficientNetB3 offers competitive classification accuracy while requiring fewer parameters. This efficiency translates to reduced demands on memory and CPU usage, making it a practical choice for achieving high performance in classification tasks without excessive computational costs.

**Figure 2:**
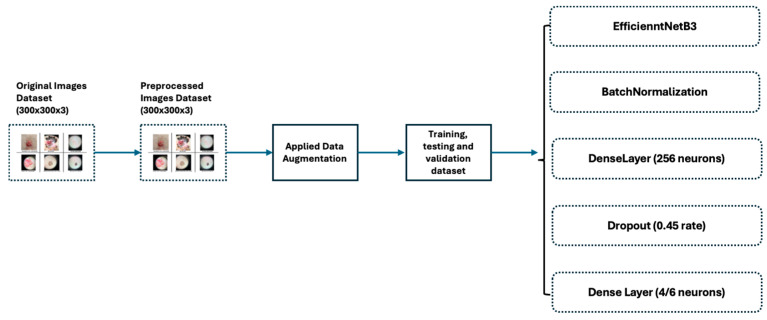
The architecture and the setup of the model.

In this study, we developed a customized model based on EfficientNetB3, using transfer learning [17,18], and leveraged ImageNet to reduce computational cost and carbon footprint. We used TensorFlow [19] and Keras [20] to integrate EfficientNet with new dense layers, allowing the model to handle complex image classification tasks. The dense layers learned high-level representations and made final predictions based on features extracted by the convolutional layers, enhancing the model’s ability to capture complex relationships and improve prediction accuracy. Fine-tuning our dataset further adapted the model to our project’s needs. The architecture of the custom model includes the added layers described below and is represented in Figure 2:

- On top of the EfficientNetB3 model, adding a batch normalization [21] layer improved accuracy by enhancing convergence and helped reduce overfitting. Batch normalization contributed to smoother training and improved generalization on unseen data by stabilizing and normalizing activations throughout the network.
- Two additional dense layers significantly enhanced classification performance by introducing non-linear features, extracting higher-level features, reducing parameter count and dimensionality of input images, and serving as a regularization technique.
- Finally, one dropout layer [22] randomly deactivated the neurons during training, which helped prevent overfitting by encouraging the model to generalize better. This technique improves the robustness and performance of the neural network on unseen data.

### 2.4. Training and Validating the Model

During this study, we applied a sum of strategies and techniques to optimize our model’s training process and performance. These included leveraging pre-trained weights from the ‘Imagenet’ dataset [14] to provide a strong starting point for learning and utilizing mixed precision policy [23] to enhance computational efficiency. We applied patience, stop patience, [24] and learning rate reduction [25] to fine-tune the training process, preventing overfitting and ensuring efficient learning rate adjustments. Transfer learning and unfreezing [26] allowed us to adapt the pre-trained model to our specific task, ensuring improved convergence and flexibility. Additionally, we saved the best weights [27] to guarantee optimal model performance for future use. Batch training [28] improved computational efficiency and promoted stable convergence through batch normalization.

#### 2.4.1. Hyperparameters

The model’s hyperparameters, listed in Table 2, fundamentally model the training process and overall performance, highlighting the importance of selecting the appropriate values.

Training a custom model for skin lesion classification using a relatively small dataset poses challenges due to noisy gradients.

We chose the Adamax optimizer [29] for its robustness against gradient fluctuations using the infinity norm, leading to stable parameter updates. This helped to improve the performance of our research on skin lesion classification, where image variability affects gradient consistency. Adamax handles maximum gradients better than SGD or Adam, supporting smoother convergence and enhanced generalization.

A learning rate of 0.001 was initially chosen and adjusted during the training as a function of the validation accuracy and loss to facilitate model convergence. The optimal batch size was set at 32, influencing convergence speed and memory demands throughout the training process. The Categorical Cross-Entropy loss and Relu activation functions were employed for multi-class classification. The model demonstrated the best performance by adopting the combination of the fine-tuned hyperparameters listed above.

#### 2.4.2. Techniques Used to Combat Overfitting

Overfitting happens when a model is overtrained on its training data, leading it to perform poorly on new data. Essentially, the model strives to be as accurate as possible, and it focuses too much on fine details and noise within its training dataset. These attributes are often not present in real-world data, so the model tends not to perform well. Overfitting can also occur when a model is too complex relative to the amount of data. This can lead the model to hyper-focus on details present in the given data that may not be relevant to the general patterns the model must develop. Overfitting gives the illusion that a model is performing well, even though it has failed to make proper generalizations about the data provided [30].

To prevent overfitting, we used several techniques that are described below:

- *Dropout*: Dropout selectively deactivates neurons in neural network layers during training, simulating smaller networks within the model. This approach encourages the network to diversify its learning strategies, enhancing generalization and mitigating overfitting by preventing reliance on individual neurons [31].
- *Batch Normalization*: Normalization adjusts data to a mean of zero and a standard deviation of one, aligning and scaling inputs. Batch normalization speeds up training by preventing gradients from becoming too small, facilitating faster convergence with higher learning rates. It also acts as a regularizer, reducing overfitting and improving model generalization on new data. This stability reduces sensitivity to initial weight choices and simplifies experimenting with different architectures [32].
- *Regularization*: We used the regularization techniques to reduce overfitting: L2 regularization with a strength of 0.016 for the kernel and L1 regularization at a strength of 0.006 for both activity and bias regularization. These methods were chosen to mitigate overfitting by penalizing large parameter values in the model, thereby promoting more straightforward and more generalized outcomes across varying datasets and scenarios.

## 3. Results

### 3.1. Training and Validation Accuracy and Loss

**Figure 3:**
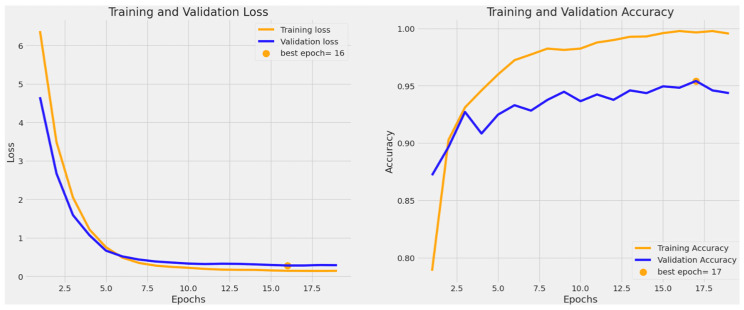
The validation accuracy and loss for the BCC, benign keratosis-like lesions. Melanocytic nevi and melanoma classes.

**Figure 4:**
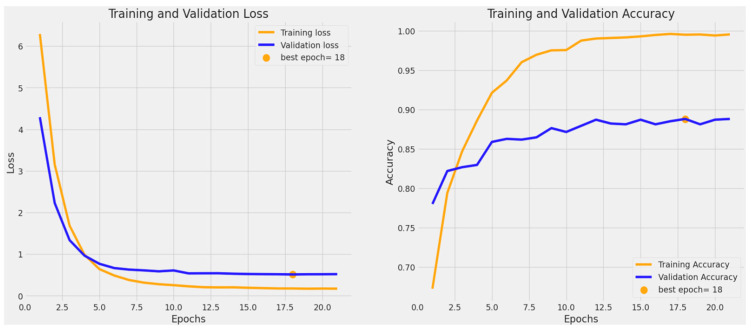
The validation accuracy and loss for BCC, benign keratosis-like lesions, melanocytic nevi, melanoma, SCC, and AK classes.

Monitoring training and validation accuracy and loss provides insights into how well a machine learning model generalizes. Lower training loss and higher accuracy indicate effective learning on seen data, while validation metrics assess performance on unseen data, ensuring the model’s robustness and generalization. Figure 3 shows an accuracy of 95.4% when our custom model was trained on four classes, whereas Figure 4 shows an accuracy of 88.8% when our custom model was trained on six classes.

### 3.2. Classification Performance

The test results show the performance metrics of the classification model, side by side, across the four different classes: BCC, benign keratosis-like lesions, melanocytic nevi, and melanoma (Table 3), and across the six different classes—the initial four, plus AK and SCC (Table 4). Performance metrics reported are precision (proportion of true positive predictions out of all positive predictions made by the model), recall (proportion of true positive predictions out of all actual positive cases in the data) and F1-score (harmonic mean of precision and recall, providing a single metric that balances both precision and recall). The model’s overall accuracy, which measures the proportion of correctly classified instances out of the total instances and provides a comprehensive evaluation of the model’s performance across all classes, is 95.4% (four classes)/88.8% (six classes).

### 3.3. Receiver Operating Characteristic (ROC) Curve

**Figure 5:**
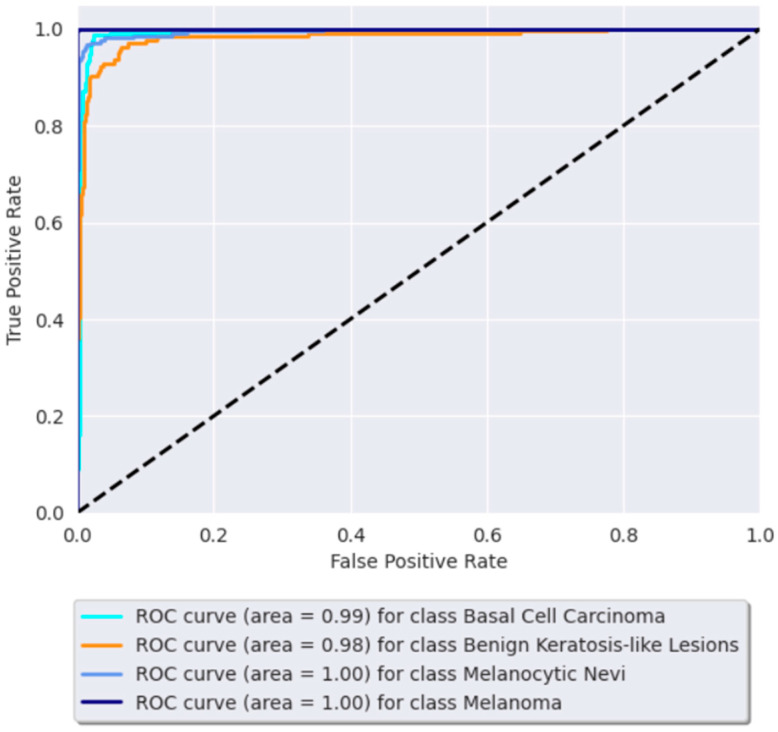
The ROC curve for the BCC, benign keratosis-like lesions, melanocytic nevi, and melanoma classes.

**Figure 6:**
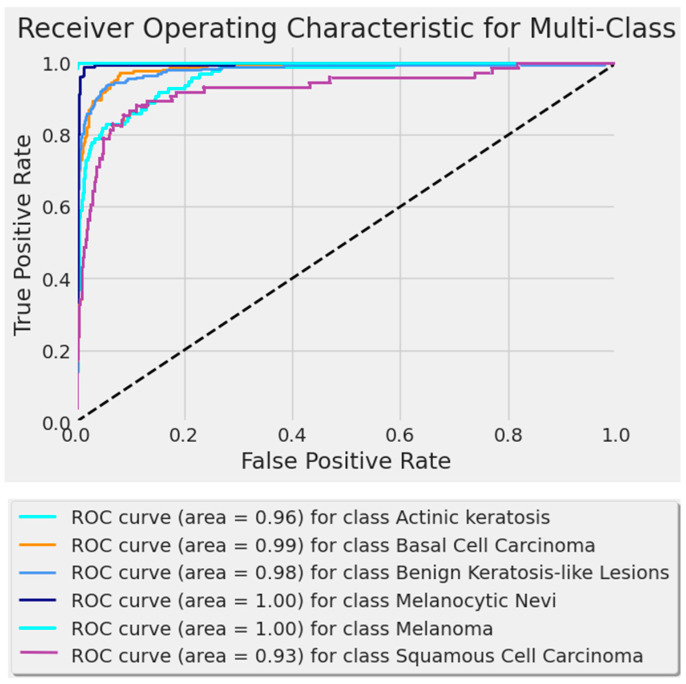
The ROC curve for BCC, benign keratosis-like lesions, melanocytic nevi, melanoma, SCC, and AK classes.

Figure 5 and Figure 6 show the proposed model’s ROC curves. The dashed line in these figures is a reference line representing a random classifier’s performance. The line, which runs from the bottom left (0, 0) to the top right (1, 1) of the ROC curve, indicates the points where the true positive rate is equal to the false positive rate. If a model’s ROC curve is above the diagonal line, the model performs better than random guessing (the further above the diagonal, the better the performance).

The curve shows excellent performance, with AUC values ranging from 0.98 to 1.0 across four classes and from 0.93 to 1 across six classes. These results are relevant to the model’s accuracy and promising clinical application potential.

### 3.4. Confusion Matrix and Errors by Class

**Figure 7:**
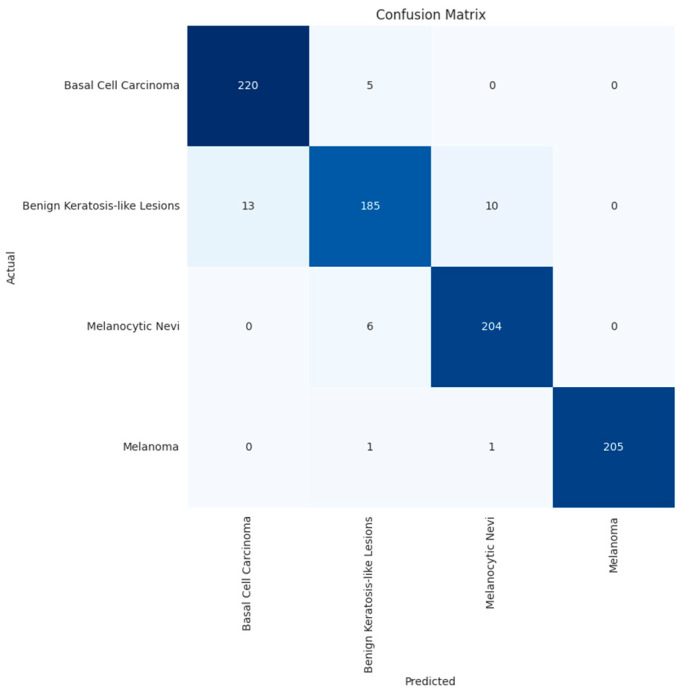
Confusion matrix for BCC, benign keratosis-like lesions, melanocytic nevi, and melanoma classes.

**Figure 8:**
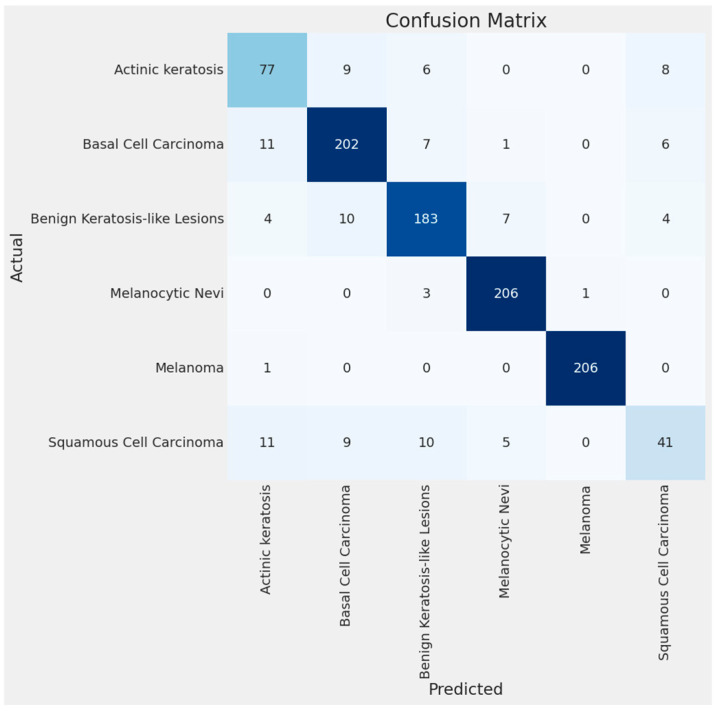
Confusion matrix for BCC, benign keratosis-like lesions, melanocytic nevi, melanoma, SCC, and AK classes.

**Figure 9:**
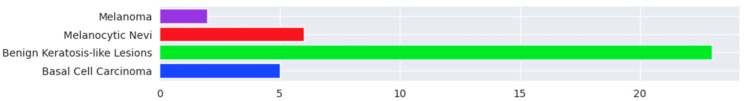
Errors per class for BCC, benign keratosis-like lesions, melanocytic nevi, and melanoma classes.

**Figure 10:**
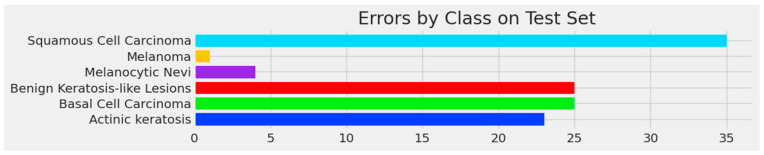
Errors per class for BCC, benign keratosis-like lesions, melanocytic nevi, melanoma, SCC, and AK classes.

Figure 7 and Figure 8 below show the confusion matrix of the custom model for the test with four and six classes, respectively. The confusion matrix visually represents the classification results of the test dataset per class. The majority of the examples per class, represented on the diagonal of the matrix, were accurately classified, i.e., for melanoma, 205 images were correctly classified out of 207. The number of errors for each class is shown in Figure 9 and Figure 10.

## 4. Discussion

AI integration in dermatology offers a substantial transformation of the medical field, and models such as EfficientNet certainly have the potential to revolutionize the diagnosis of skin lesions, providing a better tool to assist with accuracy and efficiency.

### 4.1. Model’s Performance

A key factor in the success of our EfficientNetB3-based model was the use of the ISIC 2019 archive for our image dataset, which significantly improved the model’s training and validation through its diversity and volume. This dataset includes a broad range of skin lesion images, including various types of skin conditions and lesion characteristics, thereby improving the model’s ability to generalize across different clinical scenarios and perform reliably on unseen data. However, the dataset poses a challenge due to class imbalance, with some lesion types being more prevalent than others. To address this, we employed data augmentation techniques for the minority classes during training. The ISIC 2019 archive also provides high-quality, expertly annotated images, ensuring consistency in the training and validation processes, which contributes to a more reliable model evaluation. Furthermore, using the well-recognized ISIC 2019 dataset allowed us to benchmark our model’s performance against other models in the research community, providing a common basis for evaluating key performance metrics such as accuracy, sensitivity, specificity, and AUC.

The results of our study showed that the custom model based on EfficientNetB3 has an impressive ability to classify skin lesions with high accuracy. The model’s performance was high in pathology categories well-represented by a large number of images (and therefore deep learnable data). An average accuracy of 95.4% was gained for the categories that had sufficient examples, demonstrating a high recognition and classification capacity. Yet, the introduction of pathologies with a lower number of images led the model performance to drop to 88.8%. The main factor contributing to the observed discrepancies in accuracy is the limited number of images available for the actinic keratosis and squamous cell carcinoma categories. This leads to several challenges. First, data augmentation, while helpful in increasing the apparent size of the dataset, cannot fully substitute for the diversity of real-world examples in these underrepresented categories. As a result, the model’s ability to generalize becomes limited, making it less effective at accurately identifying new, unseen instances of these lesions. Additionally, the model may develop a bias towards more represented classes, as it has more training data to learn from in those categories. Consequently, the model tends to perform better in well-represented classes and worse in underrepresented ones. Therefore, the reduced performance suggests the necessity for a balanced and representative dataset in each pathology category. These results stress the necessity to enhance data acquisition, allowing the model to be more generalizable and applied to a variety of clinical scenarios, thereby providing reliable diagnostic outcomes.

To provide a comprehensive evaluation of our proposed model, its performance was compared with the results of several studies that utilize the EfficientNet architecture for skin lesion classification. Table 5 briefly summarizes comparative studies that used EfficientNet models, with a more detailed description of this study’s work presented below.

In their study, Karthik et al. (2022) introduced Eff2Net, a model designed to classify skin diseases with improved accuracy and reduced computational complexity. By integrating the efficient channel attention (ECA) block into the EfficientNetV2 architecture, the authors replaced the traditional Squeeze and Excitation (SE) block, thereby significantly reducing the number of trainable parameters. Eff2Net was trained on a diverse dataset comprising 4930 images, with data augmentation expanding this to 17,329 images across four skin disease categories: acne, AK, melanoma, and psoriasis. The model achieved a testing accuracy of 84.70%, outperforming other contemporary models like InceptionV3, ResNet50, DenseNet-201, and EfficientNetV2 in overall accuracy with fewer parameters. Despite its strengths in reducing computational complexity and achieving high accuracy, Eff2Net has limitations, particularly in the accuracy of actinic keratosis [33].

Ali et al. (2022) explored the use of EfficientNet models (B0–B7) for classifying seven classes of skin lesions using the HAM10000 dataset. Transfer learning from pre-trained ImageNet weights and fine-tuning on the HAM10000 dataset were applied to train the EfficientNet variants. Performance metrics like precision, recall, accuracy, F1 score, specificity, ROC AUC score, and confusion matrices were used to evaluate the models. The findings revealed that intermediate complexity models, such as EfficientNetB4 and B5, performed the best, with EfficientNetB4 achieving an F1 Score of 87% and a top-1 accuracy of 87.91% [34]. It is noteworthy that the accuracy of the EfficientNetB3 model in this study was reported to be 83.9% [34], which is lower than the accuracy achieved by our proposed model.

In their study, Rafay et al. (2023) aimed to perform the classification of a wide range of skin diseases (31 categories) using a novel dataset by blending two existing datasets, Atlas Dermatology and ISIC, resulting in 4910 images. The study utilized transfer learning with three types of convolutional neural networks: EfficientNet, ResNet, and VGG, and found that EfficientNet achieved the highest testing accuracy. The EfficientNetB2 model was identified as the top performer, mainly due to its compound scaling and depth-wise separable convolutions, which enable efficient training with fewer parameters [35].

The study performed by Venugopal et al. (2023) focused on the binary classification of skin lesions (malignant vs. benign) using EfficientNet models (EfficientNetV2-M and EfficientNetB4) and a database created by combining datasets from ISIC 2018, ISIC 2019, and ISIC 2020, totaling 58,032 images. The modified EfficientNetV2-M model achieved high performance, with an accuracy of 95.49% on the ISIC 2019 dataset, while the accuracy of the EfficientNetB4 model was 93.17%. [36].

In their study, Harahap et al. (2024) investigated the use of EfficientNet models for classifying BCC, SCC, and melanoma using the ISIC 2019 dataset. The study implemented all eight EfficientNet variants (B0 to B7), with EfficientNetB4 achieving the highest overall accuracy of 79.69%. The EfficientNetB3 model achieved a validation accuracy of 74.87% and a testing accuracy of 77.60%, with a precision of 85.98%, recall of 73.44%, and F1-score of 79.21% [37]. Notably, these results are lower than the ones reported in our study, where we classified six diseases, including the three from the mentioned study, and achieved higher accuracy.

To summarize, these recent studies showcase models like EfficientNetB0, EfficientNetB2, EfficientNetV2-M, EfficientNetB4, and EfficentNetB3. The datasets used are varied, including DermNet NZ, Derm7Pt, DermatoWeb, Fitzpatrick17k, HAM10000, ISIC 2019, and proprietary collections, covering both public and private data sources. The scope of these studies is diverse, with some focusing on a broad range of skin diseases, such as 31 classes in EfficientSkinDis [35]. In contrast, others concentrate on specific categories like four to seven skin diseases, covering both benign and malignant lesions. Also, several studies tested the accuracy of EfficientNet models in comparison with other CNNs, which they surpassed in performance. The reported accuracies ranged from 84.7% to 95.49% (higher values only for binary classification), highlighting the variations in model performance depending on the dataset and classification task (binary or multi-class). Notably, the proposed model achieves 95.4% accuracy for classifying four skin diseases and 88.8% for six skin diseases, demonstrating competitive performance within this comparative framework.

Moving forward, the proposed model was also compared with other state-of-the-art classification models, all of them using images from ISIC or HAM10000 datasets (Table 6).

Several published studies focus on binary classification (benign vs. malignant) using the Kaggle/ISIC dataset [38,39,40]. More specifically, Bazgir and colleagues (2024) presented an approach to classify skin cancer using an optimized InceptionNet architecture. The study focused on distinguishing between melanoma and non-melanoma skin lesions using a dataset of 2637 dermoscopic images, split into 1197 malignant and 1440 benign lesions. The InceptionNet model was evaluated using performance metrics, including precision, sensitivity, specificity, F1-score, and area under the ROC curve. The optimized InceptionNet achieved an accuracy score of 84.39% and 85.94% when using Adam and Nadam optimizers, respectively [38]. Using the same dataset, Rahman et al. (2024) presented an approach to classify skin cancer using the NASNet architecture optimized for improved performance in detecting malignant versus benign lesions. The NASNet model’s performance was evaluated using metrics like precision, sensitivity, specificity, F1-score, and area under the ROC curve. The optimized NASNet model achieved an accuracy of 86.73% [39]. In their study, Anand et al. (2022) focused on improving the VGG16 model using transfer learning for the classification of skin cancer into benign and malignant categories. The VGG16 model was enhanced by adding a flattened layer, two dense layers with the LeakyReLU activation function, and another dense layer with the sigmoid activation function. The improved model achieved an overall accuracy of 89.09% on a batch size of 128 using the Adam optimizer over ten epochs [40].

Singh et al. (2022) introduce a novel two-stage DL pipeline named SkiNet for the diagnosis of skin lesions. The framework integrates lesion segmentation followed by classification, incorporating uncertainty estimation and explainability to enhance model reliability and clinician trust. Using Bayesian MultiResUNet for segmentation and Bayesian DenseNet-169 for classification, the SkiNet pipeline achieves a diagnostic accuracy of 73.65%, surpassing the standalone DenseNet-169’s accuracy of 70.01% [41]. Having the same image dataset and scope of classifying skin diseases into seven categories, Ahmed et al. (2024) presented a new deep learning model, SCCNet, based on the Xception architecture, with the inclusion of additional layers to enhance performance. These layers include convolutional layers for feature extraction, batch normalization layers for improved convergence, activation layers to introduce non-linearity, and dense layers for better classification performance. The model achieved an accuracy of 95.20%, with precision, recall, and F1-score values all above 95%, outperforming several state-of-the-art models such as ResNet50, InceptionV3, and Xception [42].

The article [43] by Al-Rasheed et al. presents a new approach to skin cancer classification using an ensemble of transfer learning models, specifically VGG16, ResNet50, and ResNet101. The study leverages conditional generative adversarial networks to augment the dataset, addressing class imbalance issues. The proposed models were trained on both balanced and unbalanced datasets, and their performance was evaluated using accuracy, precision, recall, and F1-score metrics. The ensemble approach achieved a superior accuracy of 93.5%, demonstrating a significant improvement over individual models, which had accuracies of around 92% [43].

Naeem et al. have published two studies using the ISIC 2019 dataset, focusing on the classification of eight types of skin diseases [44,45]. In [44], DVFNet achieved an impressive accuracy of 98.32%, outperforming several baseline CNN models like AlexNet, VGG-16, InceptionV3, and ResNet50. In [45], the proposed SNC_Net model outperforms baseline models like EfficientNetB0, MobileNetV2, DenseNet-121, and ResNet101, achieving an accuracy of 97.81%.

To sum up, the proposed model addresses a complex task of multi-class classification while still achieving an accuracy (95.4% for four classes and 88.8% for six classes) superior to the binary classification accuracies of the Inception Network, VGG16, and NASNet models. Also, the proposed EfficientNetB3 model outperforms Bayesian DenseNet-169, which achieved an accuracy of 73.65%. While SNC_Net and DVFNet achieve higher accuracies (97.81% and 98.32%, respectively), it is essential to recognize that these models benefit from more specialized architectures and additional data preprocessing techniques. Overall, the proposed model using EfficientNetB3 demonstrates strong performance in multi-class classification tasks, particularly given its simpler architecture and lower computational requirements, with its competitive accuracy highlighting EfficientNetB3’s capability to handle diverse and challenging dermatological datasets effectively.

### 4.2. Model’s Deployment and Clinical Applications

In addition to the detailed evaluation of the model’s performance and comparison with other architectures presented in this research, it is essential to address more practical aspects related to the model’s deployment and potential clinical applications. Notably, EfficientNetB3 demonstrated superior results with smaller, cheaper, and faster inference times compared to other models, which may result in a series of implications for clinical applications in dermatology. The smaller size and lower computational requirements of EfficientNetB3 reduce the cost of hardware and energy consumption, making the model more accessible for use in clinics with limited resources and potentially democratizing access to advanced diagnostic tools across various healthcare settings, including underserved regions. The EfficientNetB3 model’s speed can enhance workflow efficiency, allowing dermatologists to quickly process and analyze skin lesion images, leading to faster diagnosis, which is particularly beneficial in busy clinical environments or in teledermatology where rapid assessments are needed. Moreover, due to its smaller size and reduced computational demands, the EfficientNetB3 model can be deployed on portable devices such as smartphones and tablets, supporting point-of-care diagnostics and enabling dermatologists to use the model in various settings, including remote or rural areas where access to specialized diagnostic tools is limited. The efficient use of computational resources may allow the model to be scaled and integrated into existing healthcare systems and telemedicine platforms without the need for extensive infrastructure upgrades, ensuring that a broader range of healthcare facilities can adopt and benefit from advanced AI-driven diagnostic tools.

Deploying the model in a real-world clinical setting requires addressing several significant ethical and practical issues to ensure fairness, safety, and effectiveness. Bias and fairness are primary concerns, as DL models can perpetuate or amplify existing biases in healthcare data and decision-making. To mitigate this, it is essential to use a diverse dataset for training, conduct regular audits to detect and reduce biases, and ensure that the model performs equitably across different demographic groups. Transparency and explainability must be prioritized by providing clear explanations of how the model arrives at predictions and ensuring thorough human validation and interpretation of results. Data privacy and security are also important; strict data handling protocols, anonymization, informed consent for data use, and the implementation of the latest security measures are necessary to protect patient information.

For clinical validation and safety, rigorous testing is needed, along with ongoing monitoring of performance and safety and clear protocols to promptly alert on model recommendations. Integration with clinical workflows should complement, not replace, clinical judgment. This involves training clinicians on the appropriate use of the model, providing easy-to-use interfaces, and establishing well-documented guidelines on the model’s limitations. Regulatory compliance is important, requiring necessary approvals, maintaining audit trails and documentation, and conducting regular compliance reviews based on geography.

Ongoing evaluation and updating of the model are essential, with systems in place for tracking real-world performance, processes for model retraining and improvement, and mechanisms for quickly addressing any issues that arise. Finally, accountability requires establishing clear frameworks for who is responsible when errors occur, defining roles and responsibilities for clinicians versus AI systems, having malpractice and liability policies, and creating processes for investigating and addressing AI-related incidents. Addressing these considerations will comprehensively ensure the ethical and practical deployment of a DL model in clinical settings.

### 4.3. Limitations of Current Research

Despite the presented advancements, our study also has several limitations that need to be recognized.

First, the dataset primarily contains images of individuals from selected demographics, skin diseases, or skin phototypes, which may not represent the population’s global diversity. This aspect could limit the model’s performance when tested on other skin types and conditions, consequently affecting its generalization.

Second, the total number of images is still relatively low, particularly for infrequent lesions such as certain types of melanoma. This might lead to overfitting, where the model overperforms on training data but underperforms if applied to new unseen data.

To mitigate these constraints, the research could evolve from the current work to widen the dataset in terms of size and diversity, with the inclusion of broader skin phototypes and less common skin conditions. It will allow the development of a model that is both accurate in common conditions and applicable with high fidelity in the early detection of less common (and likely more dangerous) lesions. For a broader range of data, more international dermatology centers are required to collaborate and compile a dataset that can lead to a model that is more representative of global diagnostic applications.

Furthermore, one of the biggest areas that need to be addressed in dermatological AI research is the need for standardization when it comes to collecting images, such as using high-resolution images as the benchmark. The efficacy of ML models, like EfficientNet, is heavily dependent on the quality of the input data. Higher-resolution images obtain the finer details of dermatological conditions, which is important for accurate specification and diagnostics. Presently, the heterogeneity of imaging due to the differences in the devices used and the settings applied to collection centers remains one of the major obstacles. Creating a standard high-resolution image during the collection process would make the training data not only more uniform but also more descriptive. The standardization also would correct some of the dataset variability that was seen when models trained on mixed-quality image datasets do not perform as consistently or have reduced diagnostic performance in different clinical settings.

Future work should also focus on improving the interpretability properties of the model, offering more details related to AI’s diagnostic rationale. This would be very helpful in educational settings and would increase the model’s acceptability in clinical practice.

Also, incorporating multimodal data like the patient’s history and demographics could improve the diagnostic accuracy of the model and make it more patient-specific. If it lays the groundwork for individual dermatological assessments, then this could be the first step in aligning more with the goals of precision medicine.

## 5. Conclusions

In our study, the implementation of a custom model based on EfficientNetB3 has demonstrated substantial potential for enhancing the diagnosis of skin lesions. Our model achieved a notably high accuracy rate (95.4%/88.8%), underscoring the critical role of a comprehensive and diverse dataset. Our findings revealed that the model’s performance remains robust when there is an ample number of images for each pathology. However, there is a noticeable decline in accuracy when dealing with new or less common pathologies that have fewer representative images.

These results underscore the vital importance of ongoing efforts to expand and diversify dermatologic image datasets. Ensuring a broad, varied, and standardized dataset is essential to maintain the efficacy and applicability of AI diagnostic algorithms across a wide range of skin conditions. Continual dataset growth will support the model’s ability to generalize effectively, thereby improving diagnostic precision and reliability across diverse clinical scenarios.

## Figures and Tables

**Table 1:** Distribution of images across the skin conditions.

Classes	No. of Images	No. of Augmented Images	Total
Melanoma	1655	489	2144
BCC	1811	333	2144
Benign keratosis-like lesions	1663	481	2144
Melanocytic nevi	1686	458	2144
SCC	606	1538	2144
AK	801	1343	2144
Total	8222	4642	12,864

**Table 2:** The hyperparameters of the model.

Hyperparameters	Values
Learning Rate	0.001
Batch Size	32
Number of Epochs	19
Optimizer	Adamax
Dropout Rate	0.45
Activation Functions	Relu, Softmax
Regularization Parameters	Kernel Regularizer: L2 regularization with strength 0.016 Activity Regularizer: L1 regularization with strength 0.006 Bias Regularizer: L1 regularization with strength 0.006
Loss Function	Categorical Cross Entropy
Augmentation Techniques	Rotate, Scale, Flip, Zoom

**Table 3:** Testing results for four categories.

	Precision	Recall	F1-Score	Support
Basal cell carcinoma	0.94	0.98	0.96	225
Benign keratosis-like lesions	0.94	0.89	0.91	208
Melanocytic nevi	0.95	0.97	0.96	210
Melanoma	1.00	0.99	1.00	207
Accuracy			0.96	850
Macro Avg	0.96	0.96	0.96	850
Weighted Avg	0.96	0.96	0.96	850

**Table 4:** Testing results for six categories.

	Precision	Recall	F1-Score	Support
Actinic keratosis	0.74	0.77	0.75	100
Basal cell carcinoma	0.87	0.84	0.85	227
Benign keratosis-like lesions	0.85	0.85	0.85	208
Melanocytic nevi	0.94	0.97	0.96	210
Melanoma	1.00	1.00	1.00	207
Squamous cell carcinoma	0.69	0.54	0.61	76
Accuracy			0.89	1028
Macro Avg	0.85	0.84	0.85	1028
Weighted Avg	0.89	0.89	0.89	1028

**Table 5:** Comparative studies using EfficientNet models.

Model	Year	Dataset	Model Used	Scope	Accuracy
Karthik et al. [33]	2022	DermNet NZ, Derm7Pt, DermatoWeb, Fitzpatrick17k	EfficientNetV2, in conjunction with the efficient channel attention block	Classification of four skin diseases: acne, AK, melanoma, and psoriasis.	84.7%
Ali et al. [34]	2022	HAM10000 dataset of dermatoscopic images	EfficientNet variants (results presented refer to EfficientNet B0)	Classification of seven skin diseases	87.9%
Rafay et al. [35]	2023	Manually curated from Atlas Dermatology and SIC Dataset	Fine-tuned EfficientNetB2	Classification of 31 skin diseases	87.15%
Venugopal et al. [36]	2023	ISIC 2019 dataset	EfficientNetV2-M	Binary classification: malignant vs. benign	95.49%
Venugopal et al. [36]	2023	ISIC 2019 dataset	EfficientNetB4	Binary classification: malignant vs. benign	93.17%
Harahap et al. [37]	2024	ISIC 2019 dataset	EfficientNetB0 to EfficientNetB7 (results reported to EfficientNetB3)	Classification of three skin diseases: BCC, SCC, melanoma	77.6%
Harahap et al. [37]	2024	ISIC 2019 dataset	EfficientNetB0 to EfficientNetB7 (results reported to EfficientNetB4, the highest result obtained)	Classification of three skin diseases: BCC, SCC, melanoma	79.69%
Proposed model		ISIC 2019 and personal images collection	EfficientNetB3	Classification of four skin diseases (benign and malign)	95.4%
Proposed model		ISIC 2019 and personal images collection	EfficientNetB3	Classification of six skin diseases (benign and malign)	88.8%

**Table 6:** Comparative studies using state-of-the-art CNN models.

Model	Year	Dataset	Model Used	Scope	Accuracy
Bazgir et al. [38]	2024	Kaggle/ISIC	Inception Network	Binary classification: malign vs. benign	85.94%
Rahman et al. [39]	2024	Kaggle/ISIC	NASNet	Binary classification: malign vs. benign	86.73%
Anand et al. [40]	2022	Kaggle/ISIC	Modified VGG16 architecture	Binary classification: malign vs. benign	89.9%
Singh et al. [41]	2022	ISIC 2018	Bayesian DenseNet-169	Classification of seven skin diseases	73.65%
Ahmed et al. [42]	2024	ISIC 2018	SCCNet derived from Xpection architecture	Classification of seven skin diseases	95.2%
Al-Rasheed et al. [43]	2022	HAM10000	Combination of VGG16, ResNet50, ResNet101	Classification of seven skin diseases	93.5%
Naeem et al. [44]	2024	ISIC 2019	SNC_Net	Classification of eight skin diseases	97.81%
Naeem et al. [45]	2024	ISIC 2019	DVFNet	Classification of eight skin diseases	98.32%
Proposed model		ISIC 2019	EfficientNetB3	Classification of four skin diseases	95.4%
Proposed model		ISIC 2019	EfficientNetB3	Classification of six skin diseases	88.8%

## Data Availability

The data presented in this study are available on request from the corresponding author due to privacy reasons.
